# Editorial for the Special Issue on Miniaturized Silicon Photodetectors: New Perspectives and Applications

**DOI:** 10.3390/mi11111010

**Published:** 2020-11-17

**Authors:** Maurizio Casalino

**Affiliations:** Institute of Applied Science and Intelligent Systems “Eduardo Caianiello” (CNR), Via P. Castellino n. 141, 80131 Naples, Italy; maurizio.casalino@na.isasi.cnr.it

Silicon (Si) technologies provide an excellent platform for monolithically integrating both photonic [1] and microelectronic [2] functionalities in the same substrate. In the last few years, a variety of passive and active Si photonic and optoelectronic devices have been reported, in particular in the field of photodetection [3,4,5] where new effects and structures have been proposed. Si photodetectors (PDs) at visible wavelengths are a commercial reality, but, unfortunately, they cannot be employed in the infrared (IR) range due the Si transparence over 1100 nm. Historically, the use of germanium (Ge) grown on Si has allowed for the realization of Si-based PDs up to 1550 nm [6]. In recent years, impressive progresses have been achieved by extending the operation of Si PDs to the infrared regime and, recently, thanks to the investigation of new smart structures and effects, the all-Si approach has demonstrated potentialities leading to performances close to those of the well-established germanium (Ge) technology. Moreover, the possibility to integrate new emerging 3D and 2D materials with Si, together with the capability of manufacturing devices at the nanometric scale, has led to the development of new devices with unexpected performance.

There are eight papers published in this Special Issue, six original works and two review articles. The spectral range covered by these works goes from the ultraviolet (UV) to the infrared (IR) regime. Among the original works, four of them are focused on the investigation of novel Si-based PDs while the remaining two on the characterization of materials that could be employed in Si technology. The four papers proposing original devices are based on Schottky, metal–semiconductor–metal (MSM) and P/N structures. The proposed Schottky photodetectors take advantage of the integration of graphene on both crystalline silicon (c-Si) and polycrystalline silicon (poly-Si) substrates while the MSM PDs are based on germanium–tin (GeSn) layers. Both structures (Schottky and MSM) have shown the capability to detect near infrared (NIR) wavelengths. On the other hand, a complementary metal–oxide–semiconductor (CMOS) single photon avalanche P/N diode has been investigated for detecting light in the visible regime. Concerning the two papers dealing with the material characterization, they investigate layers of 4H-SiC and ZnO to be integrated on Si for detecting UV and visible regime. Finally, two review articles are focused on the development of CMOS-compatible microbolometers and integrated PDs based on group IV and colloidal semiconductors.

In particular, Tsai et al. [7] proposed a graphene/poly-Si PD to monolithically integrate with the electronic circuitry constituting the active pixel of a CMOS image sensor. This work is interesting mainly for two reasons. First, although graphene/crystalline Si PDs have been frequently reported in the literature [8], the investigation of graphene/polycrystalline junctions is much less frequently discussed. Second, the use of polycrystalline silicon as semiconductor, instead of crystalline silicon, makes the photodetector able to be directly integrated on top of the gate oxide of a conventional metal–oxide–semiconductor field effect transistor (MOSFET), enabling the realization of a compact active pixel to be employed in CMOS image sensors. The authors name this new proposed structure as: photodiode–oxide–semiconductor field effect transistor (PDOSFET). If the device described in the work of Tsai et al. should operate in the visible range, M. Casalino theoretically investigates the possibility of employing hybrid graphene/c-Si Schottky diodes to detect NIR wavelengths [9]. In this work, the absorption mechanism is based on the internal photoemission effect: graphene first absorbs the incoming radiation and then it transfers the photoexcited carriers into Si where they are collected. In addition, this work suggests integrating the graphene layer in the middle of a silicon-based Fabry–Pérot microcavity constituted by an amorphous hydrogenated silicon/graphene/crystalline silicon three-layer system surrounded by two high reflectivity mirrors. The author shows that the enhancement of the optical field inside the cavity allows a significant increase in graphene optical absorption and, consequently, in device efficiency. Theoretical results show responsivity of 0.24 A/W, bandwidth in GHz regime, noise equivalent power of 0.6 nW/cm√ Hz. MSM PDs have been proposed by Ghosh et al. taking advantage of GeSn layers integrated on Ge-buffered Si substrates for short-wave infrared (SWIR) applications [10]. Indeed, GeSn shows a significant absorption along the entire telecommunication bands unlike germanium (Ge), whose optical absorption falls drastically beyond 1550 nm. GeSn MSM PDs have been both electrically and electro-optically characterized. The I–V electrical characteristic shows the classical MSM behavior while the spectral responsivity measurements show a broadband optical absorption extending over 1800 nm. The reported responsivity increases by increasing the bias voltage and at 7V maximum values of about 100, 70 and 10 mA/W at 1200, 1500 and 1800 nm, have been reported, respectively. The paper of Goll et al. investigates the discharge mechanism of single-photon avalanche diodes (SPADs) designed in 0.35 μm CMOS technology [2]. Indeed, after the avalanche has been triggered, the SPAD cathode–anode voltage reaches the breakdown voltage with a time that has been measured by the authors. Based on the cathode capacitance measurements, the avalanche current through each SPAD was evaluated too. Measurements on the cathode voltage transient of SPADs based on a 12 μm-thick p^-^ epi-layer (named type A) with various diameters were investigated. Results show fall times of 3.45 ns for 200 μm diameter SPAD and an excess bias (voltage difference between diode work reverse voltage and the breakdown voltage) of 4.26 V, as well as fall times of 10.2 ns for 50 µm diameter SPAD and an excess bias of 4.2 V. On the other hand, SPADs with different diameters were implemented in the high-volume (HV) line of the same CMOS process (named type B) showing fall times of 2 ns for 98.2 µm diameter SPAD and 5.9 V excess bias, as well as 8 ns for 48.2 µm diameter SPAD and 5.4 V excess bias.

Moving our attention onto the characterization of materials to be employed for the realization of Si-based photodetectors, J. Li et al. investigate how different chemical vapor deposition (CVD) growth conditions impact the defect density of 4H-SiC epilayers and the performance of Nickel(Ni)/4H-SiC Shottky PDs [11]. In this work, particular attention was paid to triangular defects (TDs) and deep level defects, Z_1/2_, showing that, while the C/Si ratio strongly impacts the formation of TDs, no correlation with the deep level defect, Z_1/2_, can be confirmed. This work shows that, by adjusting the C/Si ratio, the quality of the 4H-SiC epilayer can be improved and the performance of the Ni/4H-SiC Schottky detector increased. In their work, G. Li et al. evaluate the impact ionization coefficient of electrons in a ZnO layer along the (001) direction [12]. In order to do it, the authors have investigated p-Si/i-ZnO/n-AZO structures illuminated by a 532 nm laser diode where the electron avalanche multiplication is triggered in the i-ZnO layer whose thickness was varied from 250 to 750 nm. These insights could be very useful for the realization of high-performance ultraviolet (UV) avalanche photodiodes).

Finally, this Special Issue includes also two review articles: Dardano and Ferrara have reported recent advances in the field of photodetection based on group IV materials with particular reference to silicon [13]. In recent years, the challenge to make silicon usable at NIR wavelengths has attracted much interest and many absorption mechanisms have been both proposed and investigated. The authors show as the mid-bandgap absorption (MBA), i.e., the infrared absorption obtained by voluntarily introducing defects in the Si bandgap, combined with high Q-factor cavity structures (ring resonators), has emerged as a viable solution for the realization of devices whose performance are comparable with the well-established Ge technology. Then, in their work, the authors reviewed PDs based on different materials, such as graphene, Ge and carbon nanotubes (CNTs). Moreover, an overview on PDs based on colloidal semiconductors, representing the frontier of future research, has been presented too. Finally, Yu et al. have reviewed the CMOS microbolometer technology for long-wave infrared (LWIR) imaging applications at a low cost [14]. This technology is based on a standard CMOS process combined with a simple post-CMOS micro-electro-mechanical system (MEMS) process. In their work, the authors show that the performance of the reported CMOS-compatible microbolometers has started to compare favorably with the state of the art. This paper reviews not only the recent advances of the CMOS-compatible microbolometers but also the aspects of the pixel structure and of the read-out integrated circuitry.

I would like to take this opportunity to thank all the authors for submitting their papers to this Special Issue. I would also like to thank all the reviewers for dedicating their time and helping to improve the quality of the submitted papers. Finally, I would like to warmly thank Ms. Aria Zeng for her constant support in preparing this Special Issue.
